# ERNICA guidelines for the management of rectosigmoid Hirschsprung’s disease

**DOI:** 10.1186/s13023-020-01362-3

**Published:** 2020-06-25

**Authors:** Kristiina Kyrklund, Cornelius E. J. Sloots, Ivo de Blaauw, Kristin Bjørnland, Udo Rolle, Duccio Cavalieri, Paola Francalanci, Fabio Fusaro, Annette Lemli, Nicole Schwarzer, Francesco Fascetti-Leon, Nikhil Thapar, Lars Søndergaard Johansen, Dominique Berrebi, Jean-Pierre Hugot, Célia Crétolle, Alice S. Brooks, Robert M. Hofstra, Tomas Wester, Mikko P. Pakarinen

**Affiliations:** 1 Department of Pediatric Surgery, Children’s Hospital, University of Helsinki and Helsinki University Hospital, Helsinki, Finland; 2 Department of Pediatric Surgery, Erasmus MC – Sophia Children’s Hospital, Rotterdam, The Netherlands; 3 Department of Surgery, Division of Pediatric Surgery, Radboudumc-Amalia Children’s Hospital, Nijmegen, The Netherlands; 4 Department of Pediatric Surgery, Oslo University Hospital and University of Oslo, Oslo, Norway; 5 Department of Pediatric Surgery and Pediatric Urology, University Hospital Frankfurt, Frankfurt/M, Germany; 6 Department of Biology, University of Florence, A.Mor.Hi, The Italian Association for Hirschsprung’s disease, Florence, Italy; 7 Pathology Unit, Bambino Gesù Children’s Hospital, IRCSS, Rome, Italy; 8 Neonatal Surgery Unit – Department of Medical and Surgical Neonatology, Bambino Gesù Children’s Hospital, IRCSS, Rome, Italy; 9 SoMA, The German patient support organization for anorectal malformations and Hirschsprung Disease, Munich, Germany; 10 Pediatric Surgery, Department of Women’s and Children’s Health, University Hospital of Padua, Padua, Italy; 11 UCL Great Ormond Street Institute of Child Health; Department of Pediatric Gastroenterology, Great Ormond Street Hospital for Children, London, UK; 12 Department of Surgery and Transplantation, Abdominal Centre, Rigshospitalet, Copenhagen, Denmark; 13 Department of Pediatric Pathology, Hôpital Universitaire Robert Debré, Paris Diderot University, Paris, France; 14 Department of Pediatric Gastroenterology, Hôpital Universitaire Robert Debré, Assistance Publique Hôpitaux de Paris, Université de Paris, Paris, France; 15 Department of Pediatric Surgery, University Hospital Necker-Enfants Malades, APHP centre, Paris University, Paris, France; 16 Department of Clinical Genetics, Erasmus Medical Center, Rotterdam, The Netherlands; 17 Department of Pediatric Surgery, Karolinska University Hospital, Stockholm, Sweden

**Keywords:** Rectosigmoid Hirschsprung’s disease, HSCR, Diagnosis, Management, Follow-up

## Abstract

**Background:**

Hirschsprung’s disease (HSCR) is a serious congenital bowel disorder with a prevalence of 1/5000. Currently, there is a lack of systematically developed guidelines to assist clinical decision-making regarding diagnostics and management.

**Aims:**

This guideline aims to cover the diagnostics and management of rectosigmoid HSCR up to adulthood. It aims to describe the preferred approach of ERNICA, the European Reference Network for rare inherited and congenital digestive disorders.

**Methods:**

Recommendations within key topics covering the care pathway for rectosigmoid HSCR were developed by an international workgroup of experts from 8 European countries within ERNICA European Reference Network from the disciplines of surgery, medicine, histopathology, microbiology, genetics, and patient organization representatives. Recommendation statements were based on a comprehensive review of the available literature and expert consensus. AGREE II and GRADE approaches were used during development. Evidence levels and levels of agreement are noted.

**Results:**

Thirty-three statements within 9 key areas were generated. Most recommendations were based on expert opinion.

**Conclusion:**

In rare or low-prevalence diseases such as HSCR, there remains limited availability of high-quality clinical evidence. Consensus-based guidelines for care are presented.

## Background

Hirschprung’s disease (HSCR) is a congenital intestinal motility disorder with an incidence of 1:5000, with a male to female predominance of 4:1. It is characterized by an absence of enteric ganglion cells from the distal intestine, causing chronic functional bowel obstruction [1]. Although HSCR can affect any length of bowel from the anus proximally, 80–85% of cases are limited to the rectosigmoid colon [2]. Operative management involves resection of the abnormally innervated bowel. Despite surgical treatment, postoperative defects in bowel function are common, and highly specialist long-term aftercare that includes transition to adult care providers is required. Currently, there is a lack of systematically developed guidelines to assist clinical decision-making in HSCR, although standardization of diagnostics, treatments and care pathways would clearly benefit patients.

Addressing healthcare inequalities and ensuring delivery of high-quality care for patients with rare or low-prevalence, complex diseases has been identified as an important target at the level of the European Union (EU) [3]. To address the geographical scattering of expert healthcare providers and patients, European Reference Networks (ERNs) for were founded. These currently comprise 24 ERNs from over 900 highly specialized healthcare units and 313 hospitals [3]. ERNICA is the ERN for rare inherited and congenital digestive disorders, including Hirschsprung’s disease. This document aims to describe the preferred approach of ERNICA for the diagnostics and management of rectosigmoid HSCR up to adulthood. A core aim of ERNICA is to improve the quality of care that patients receive, and to reduce the long-term impact of HSCR for patients [3].

## Results

### Recommendations for the diagnosis of HSCR (Table 1)

Around 90% of patients with HSCR present during the neonatal period [4]. In rectosigmoid HSCR, there is a male predominance of 4:1 [5]. The classic clinical symptoms include abdominal distension (> 90%), vomiting (> 85%), which may be bilious, and failure to pass meconium during the first 24 h of life (> 60%) [4]. Digital rectal examination or passage of a rectal tube typically results in the evacuation of gas and faeces, which may be explosive and/or foul-smelling. Plain abdominal x-ray findings include dilated gas-filled loops of bowel suggestive of a distal obstruction. Some patients may have symptoms of enterocolitis at presentation. A family history of HSCR or presence of a syndrome associated with HSCR lowers the threshold for investigation. According to a recent systematic review, only 5% of 4127 infants diagnosed with HSCR between 1964 and 2013 were premature (< 37 weeks’s gestation), but this proportion was higher (14%) amongst patients born during recent years [6]. Contemporary studies suggest that late presentation beyond 3 years of age is unusual [4, 7].

**Table 1:** Recommendations for the diagnosis of HSCR

**The diagnosis of HSCR should be based on representative rectal histology, and should be confirmed before pull-through surgery.** • Rectal suction biopsy (RSB) and open biopsy are equally accurate if they provide enough submucosal tissue. The least invasive, feasible method should be chosen. • Biopsies should be taken from the posterior and/or lateral rectal wall at least 2 cm proximal to the dentate line or 3 cm from the anal orifice, and must contain a representative amount of submucosa. • A minimum of 1 histologically representative tissue sample is required. One open biopsy usually provides sufficient tissue but with RSB it is advisable to take 2–3 biopsies.	Level of evidence III Strength of recommendation: Strong, *for* Level of agreement: 100%
**Rectal biopsy is indicated if the clinical history and physical signs are suggestive of HSCR.** • The classic triad of symptoms is delayed passage of meconium (> 24 h in a term infant), abdominal distension and bilious vomiting. • The majority of patients present during the neonatal period or early infancy**.** • The threshold for biopsy is lowered by the presence of a syndrome associated with HSCR or family history of HSCR	Level of evidence III Strength of recommendation: Strong, *for* Level of agreement: 100%
**Rectal biopsy should also be considered for the exclusion of HSCR in:** • Early-onset constipation associated with failure to thrive • Older children with persistent constipation or symptoms of more generalized intestinal motility disorders • Patients with an absent recto-anal inhibitory reflex (RAIR) on anorectal manometry	Level of evidence III Strength of recommendation: Conditional, *for* Level of agreement: 100%
**Biopsies should be evaluated by an experienced consultant histopathologist, seeking external consultation if necessary** • The presence of any number of ganglion cells on hematoxylin and eosin (H&E) staining excludes HSCR. • If ganglion cells are not seen, additional histologic evaluation should be considered before setting a diagnosis of HSCR. • Calretinin and/or peripherin should be used to look for ganglion cells, particularly in premature infants where these are small and not well visualised on H&E. • In HSCR, acetylcholinesterase activity is increased, and calretinin immunohistochemistry is negative. (Within Europe, external consultation can be requested from an ERNICA centre. A Clinical Patient Management System e-platform is under development)	Level of evidence III Strength of recommendation: Strong, *for* Level of agreement: 100%

Representative rectal histology is required for the diagnosis of HSCR. Rectal biopsies may be taken as suction or open surgical biopsies, opting for the least invasive, feasible method. Biopsies should be taken a minimum of 2 cm above the dentate line to avoid the physiologic aganglionic/hypoganglionic zone of the distal rectum [8]. The International Working Group of the 2009 World Congress of Gastroenterology advocates that a biopsy specimen should be at least 3 mm diameter, and a minimum of one-third of the sample should comprise submucosa [9, 10]. Hematoxylin-eosin (H&E) staining and Acetylcholine-Esterase (AChE) histochemistry are widely used. The presence of any number of ganglion cells on H&E staining excludes HSCR. However, H&E has limitations for the visualisation of ganglion cells, particularly in neonates and premature babies, where these may be small and immature. If ganglion cells are not seen, additional staining with calretinin and/or peripherin immunohistochemistry is advisable before setting the diagnosis of HSCR [11, 12]. Increased AChE expression is associated with the hypertrophied extrinsic nerve fibres of the aganglionic segment in most patients with HSCR [13]. With AChE, false negatives are primarily related to age, and absence of an AChE reaction does not reliably exclude HSCR in very young neonates [13, 14]. The sensitivity and specificity of other methods, including anorectal manometry and contrast enema are inferior to those of an adequate rectal biopsy, particularly in young infants [15, 16]. The histopathologic assessment of HSCR is demanding, as it remains centered around demonstrating an absence of ganglion cells [8]. Careful evaluation by an experienced consultant histopathologist is necessary, using a combination of staining techniques whenever possible. External consultation is recommended in unclear cases.

### Recommendations for who should operate on patients with HSCR (Table 2)

The ERNICA recommendations for centres performing pull-through surgery for HSCR are in accordance with recent European recommendations concerning the management of patients with rare diseases [17]. There is reliable evidence that performing the definitive surgical management of rare pediatric neonatal surgical conditions in specialist units improves treatment outcomes and safety for several reasons [18–20]. The concentration of care in cases with an otherwise low prevalence permits care based on sufficient cumulative experience of the indications for surgery, surgical procedures, perioperative management and post-operative follow-up [21]. The presence of allied medical specialists, including neonatal intensivists, nurses, and out-of-hours emergency services with experience in the condition also improves the “rescue phenomenon,” which refers to the ability to prevent minor postoperative events from escalating into severe complications and mortality [21, 22]. At index centers, the need for re-do pull-through operations is low, with mostly good to normal long-term continence outcomes in rectosigmoid HSCR [2, 23, 24]. From economic and policy-planning standpoints, the prevention of severe complications is also the most important determinant of the cost-effectiveness of care [25].

**Table 2:** Recommendations for who should operate on patients with HSCR

**Patients with HSCR should undergo pull-through surgery in centres with at least two pediatric colorectal surgeons and pathological, radiological and anesthetic expertise, including pediatric and neonatal intensive care and specialized nursing 24/7.** • Concentration of interdisciplinary experience is associated with better outcomes in complex or rare pediatric surgical conditions. • Accurate primary assessment of the disease phenotype in HSCR permits appropriate surgical management • The need for re-do surgery is low in centres that regularly manage HSCR and complications are appropriately identified and managed	Level of evidence III Strength of recommendation: Strong, *for* Level of agreement: 100%
**Centres performing pull-through surgery for HSCR should have the capabilities to manage the entire care pathway** • This includes management of surgical complications and primary surgical management of all forms of HSCR, provision of multidisciplinary care until adulthood, and specialist nursing • Ability to deliver comprehensive follow-up until adulthood, including provision of transition of care	Level of evidence III Strength of recommendation: Strong, *for* Level of agreement: 100%
**Centres that operate on HSCR patients should demonstrate active involvement in quality control and improvement** • Maintaining prospective registries permits assessment and monitoring case volumes and outcomes • Commitment to training surgeons, pathologists and nurse practitioners in diagnostics and management of HSCR ensures continuity of local expertise • Up-to-date care practices and understanding of the disease process through networking and participation in continued medical and surgical education • Information should be given about the availability of patient support organizations as early as possible, and patients should be informed about the availability of current guidelines	Level of evidence III Strength of recommendation: Strong, *for* Level of agreement: 100%

On a national level, favouring the feasibility of organized care networks for HSCR is the finding that patients are willing to travel long distances in order to even marginally reduce their risk of complications and death, and for better access to multidisciplinary services [21, 26]. Registries of care volumes, outcomes, cost of care and service infrastructure are necessary to enable patients, healthcare professionals and policy-makers to make informed decisions regarding the choice of care provider. Having at least two pediatric colorectal surgeons within units assists to ensure the availability of a year-round surgical service for HSCR, and the option of two-consultant operating in complex cases. Commitment to training surgeons and other allied specialists ensures long-term service continuity.

Centres performing pull-through surgery for HSCR should have the capacity to manage all levels of HSCR, as aganglionosis extending proximal to the rectosigmoid affects 15–20% of patients [23]. Despite an optimal pull-through, impairment of bowel function and enterocolitis are common during the first few years after surgery, and between 4 and 30% of patients have an associated developmental disorder or syndrome [23, 27, 28]. Competence in the management of HSCR is therefore not only defined by an ability to perform complex surgery or hospital volumes alone, but also on capacity to manage the disease process as a whole and to provide individualized long-term follow-up into adulthood [21]. For this, a properly functioning multidisciplinary approach is essential [21]. Evidence of quality should be based on outcome registries, alongside regular participation in internal and external assessment of benchmarks. Involvement in research is beneficial for generating knowledge regarding the effects of current and competitive therapies, which are needed to develop practices towards new standards.

### Recommendations for preoperative care (Table 3)

Whilst awaiting the results of the rectal biopsy, saline rectal irrigations should be commenced to overcome the functional bowel obstruction and to enable enteral feeding. Parents can be trained to continue them at home on a daily basis until the pull-through operation. Rectal irrigations with physiological saline are performed with a soft rectal tube one to three times a day. Rectal irrigations provide effective bowel decompression in approximately 75% of HSCR patients. Patients in whom rectal irrigations fail often turn out to have long- or extended segment aganglianosis [2]. In these cases or if there is enterocolitis unresponsive to non-operative treatment, or intestinal perforation, a diverting enterostomy is indicated [29]. The safest empiric level is the distal ileum, based on the likelihood that aganglionosis will be confined distally to the colon. Whenever possible, frozen section biopsies at the planned stoma site should be obtained to confirm the presence of ganglion cells. A circumferential full thickness ‘doughnut’ biopsy from the site of bowel transection is most informative regarding the ganglion status at that level [29]. Additional full-thickness or seromuscular mapping biopsies may be obtained during the same operation to define the exact level of the histologic transition zone for planning the definitive pull-through surgery [30].

**Table 3:** Recommendations for preoperative care in HSCR

**Patients should receive saline rectal irrigations 1–3 times per day to decompress the bowel until the definitive pull-through operation** • An additional colonic wash-out may be given for pre-operative bowel preparation • See below, if there is an inadequate response to rectal irrigations	Level of evidence III Strength of recommendation: Strong, *for* Level of agreement: 100%
**A stoma is indicated if rectal irrigations do not sufficiently decompress the bowel, or there are complications such as enterocolitis unresponsive to non-operative treatment, or bowel perforation.** • The safest empiric level is an ileostomy • In pneumoperitoneum, also an ileostomy provided it is proximal to the site of perforation • A representative circumferential ‘doughnut’ biopsy taken from the site of stoma formation is informative regarding the ganglionic status of the bowel at that level	Level of evidence III Strength of recommendation: Strong, *for* Level of agreement: 100%
**When possible, a pre-operative contrast enema is recommended to guide on the likely level of aganglianosis** • A colonic caliber change suggests a histological transition zone at this level. • Proximal to the rectosigmoid junction, colonic caliber changes are less accurate in predicting the disease level, and the possibility of long-segment HSCR should be considered • Contrast studies are complementary tools during the pre-operative workup. They do not replace the need for histological assessment to confirm the diagnosis.	Level of evidence III Strength of recommendation: Conditional, *for* Level of agreement: 100%
**At pull-through surgery, one dose of broad-spectrum intravenous antibiotics should be given preoperatively.** • The choice of antibiotics is determined by local regimens and regional resistance profiles, but should include coverage of both aerobic and anaerobic bacteria • No additional benefit has been shown for giving more than one pre-operative dose, but antibiotics may be continued for 24–48 h post-operatively	Level of evidence II-III Strength of recommendation: Strong, *for* Level of agreement: 100%

In a recent European survey, 96% of responders performed a pre-operative contrast enema to guide on the likely level of aganglionosis [31]. Although a contrast enema is not considered sufficient on its own for the diagnosis of HSCR, a distinct calibre change from proximally dilated to distal constantly narrow colon suggests a transition zone at this level. The most demonstrative images are often obtained just after evacuation of the contrast material. If a contrast enema examination fails to suggest a clear transition zone in the recto-sigmoid colon, the possibility of long-segment aganglionosis and additional mapping biopsies should be considered [30].

In adult colorectal surgery, administration of a single preoperative dose of antibiotics has been shown to reduce postoperative wound infections by 75% [32]. A combination of aerobic and anaerobic bacteria coverage with a single agent or combination therapy is most effective, the choice depending on local resistance patterns. Administration of prolonged antibiotic prophylaxis has not been proven to be more effective in preventing surgical wound infections [33]. It is unknown to what extent this reduces sequelae from anastomotic leakage, deep surgical infections, urinary tract or respiratory infections [32]. Studies of antibiotic prophylaxis in the pediatric surgical population are scarce and do not include the latest techniques for HSCR surgery using minimally invasive or transanal approaches [33].

### Recommendations for operative management of rectosigmoid HSCR (Table 4)

Endorectal pull-through (ERP) and Duhamel pull-through are the most common definitive operations for HSCR [31, 34]. Both ERP and Duhamel can be performed laparoscopy- or laparotomy assisted, although ERP can also be performed entirely transanally in rectosigmoid HSCR [35–38]. Currently, there is no evidence to favor superiority of one technique over another in terms of surgical complications or long-term bowel functional outcomes [39–43]. Pull-through surgery is usually performed electively within 2–3 months after diagnosis and when the infant is stable, growing well and the bowel has been decompressed. No specific advantages have been identified for performing pull-through surgery during the immediate neonatal period. However, there remains insufficient data for establishing the optimal timing of pull-through surgery.

**Table 4:** Recommendations for operative management of rectosigmoid HSCR

**Centres should perform the type of pull-through in which they have the most experience, including management of post-operative complications and follow-up** • Transanal endorectal pull-through (ERP), including laparoscopy-assisted ERP, and Duhamel pull-through represent the most commonly performed operations. Currently, there is no evidence for overall superiority of one method over another in terms of surgical complications or long-term bowel function.	Level of evidence III Strength of recommendation: Conditional, *for* Level of agreement: 100%
**The pull-through operation should be performed when the patient is stable and growing well, and the bowel has been sufficiently decompressed** • Elective pull-through within 2–3 months after diagnosis is usual • No specific advantages have been identified for performing pull-through surgery during the neonatal period • Anaesthetic considerations, clinical and nutritional status of the patient, parental concerns and surgical risks/technical feasibility influence the timing of pull-through surgery	Level of evidence III Strength of recommendation: Conditional, *for* Level of agreement: 100%
**The anal canal should be preserved during pull-through surgery** • The transitional mucosa above the dentate line contains the nerve endings responsible for the reflex arc in sensation and fecal continence, including the sampling reflex. • Transanal dissection should be commenced 0.5–2 cm proximal to the dentate line • In endorectal pull-through, either no muscle cuff (Swenson) or a short muscle cuff (< 2-3 cm) have comparable outcomes, but long seromuscular cuffs should be avoided	Level of evidence II-III Strength of recommendation: Strong, *for* Level of agreement: 100%
**The colon should be transected at least 5 to 10 cm proximal to the first normal biopsy minimize the risk of a transition zone pull-through.** • If the level of disease remains intraoperatively uncertain, ‘mapping’ biopsies should be obtained from different colonic levels. • Intraoperatively, fresh frozen sections are a valid means for determining the presence of normal ganglionated bowel but single samples may miss asymmetrical histologic extension of the transition zone. • A circular ‘doughnut’ biopsy from the level of transection permits circumferential (4-quadrant) optimal histologic assessment • If feasible, any abnormally dilated colon proximally should be resected to avoid a transition zone pull-through. • At the end of the operation, the resected specimen should be sent in full (marked oral to anal) to the pathologist.	Level of evidence III Strength of recommendation: Strong, *for* Level of agreement: 100%

Based on case series, it remains debated whether laparoscopy- or laparotomy assisted ERP results in less stretching of the anal sphincters than a totally transanally performed ERP [28, 44, 45]. In Duhamel operation, no difference in bowel functional outcomes between laparoscopic and open approaches has been shown [38, 46]. There are also no data regarding the optimal length of the Duhamel pouch, and if modifications of the retro-rectal anastomosis reduce the need for spur divisions [38]. Although the Duhamel operation leaves a distal segment of aganglionic bowel anteriorly, it causes very little anal stretching and affords good visibility during surgery. A totally transanal ERP, on the other hand, confers excellent cosmesis [46]. The original transanal ERP described a long muscular rectal cuff that was split [47–50]. Later reports showed equally good results with a short muscle cuff without splitting, and no cuff with full-thickness plane dissection [44, 48, 51–53]. Based on current understanding, long seromuscular cuffs should be avoided as they are associated with obstruction, constipation, and enterocolitis [46]. Preserving the integrity of the anal canal is a key goal during the operative management of all forms of HSCR. The mucosa above the dentate line contains the nerve endings responsible for the reflex arc in sensation and fecal continence, including the sampling reflex. In ERP, transanal dissection is commenced 0.5–2 cm proximal to the dentate line and in the Duhamel operation the posterior incision is performed 0,5-1 cm above it [47, 49, 51, 54].

No definite pull-through surgery should be performed without first establishing that normal ganglion cells are present in the bowel brought down to the anal canal for anastomosis. During surgery, frozen sections are a valid means for determining the presence of ganglion cells. Full-thickness biopsies permit examination of both the myenteric and submucosal plexuses and thereby minimize the chance of anastomosing abnormally innervated bowel to the anal canal. Seromuscular biopsies have a lower risk of fecal spillage and postoperative perforations, but may be more prone to sampling errors if the transition zone is irregular due to a lack of full submucosal assessment [46]. As the length of the transition zone may be variable, the proximal transection margin should be a minimum of 5–10 cm orally to where normal ganglion cells are found, unless a “doughnut” biopsy, showing ganglion cells circumferentially in both the submucosal and myenteric plexus is available intraoperatively [55].

### Recommendations for early postoperative management after pull-through surgery (Table 5)

Enhanced recovery after surgery (ERAS) protocols, which have improved outcomes in adult surgical populations were recently adapted for children undergoing elective colorectal surgery [56]. ERAS aims to optimize care in major surgical procedures through maintenance of physiologic homeostasis and by minimizing the effects of surgical stress. In children undergoing colorectal surgery, the median the time to regular diet, requirement for narcotic analgesia requirement and length of hospital stay were significantly reduced [56]. General tenets of ERAS include avoidance of prolonged fasting, use of minimally invasive surgical techniques, opioid-sparing analgesia, early reinstitution of enteral nutrition and judicious use of drains and catheters [56]. A critical feature involves engagement of the patient and carers at all phases of care. This is conducted by creating clear goals, establishing management plans for pain and nutrition, and defining the criteria for discharge to facilitate an optimal surgical experience [57].

**Table 5:** Recommendations for early postoperative management after pull-through surgery

**Patients should receive specialist pediatric and nursing care during the early post-operative period, and anaesthetic consultation should be available on request** • Use of Enhanced Recovery After Surgery (ERAS) [56] principles may reduce length of stay, requirement for narcotic analgesia and time to full enteral feeds • Parental counselling is important to ensure understanding and engagement with the care plan • Once bowel movements begin, perianal rash/skin excoriation is initially common and requires pre-emptive nursing	Level of evidence III Strength of recommendation: Conditional, *for* Level of agreement: 100%
**Enteral feeding can be started gradually when the patient has recovered from anaesthesia and is clinically stable** • Within 24–48 h in most cases • Advance feeds as tolerated to normal diet • There is no evidence to suggest prolonged nil by mouth periods or prevent anastomotic complications	Level of evidence III Strength of recommendation: Conditional, *for* Level of agreement: 100%
**The urinary catheter should be removed as soon as normal micturition is expected after pelvic floor surgery** • Epidural anaesthesia post-operatively is an indication for keeping a urinary catheter • Urinary retention after removal can occur following anaesthesia or post-operative tissue swelling in the pelvic floor, and adequacy of urine output should initially be monitored.	Level of evidence III Strength of recommendation: Conditional, *for* Level of agreement: 100%
**The coloanal anastomosis should be calibrated around 2–3 weeks after pull-through surgery** • Hegar size 12 is appropriate for infants from term up to 6 months of age • Routine serial dilatations have not been shown to reduce the prevalence of enterocolitis or late anastomotic strictures. • If an anastomotic stricture is found, a course of gentle serial dilatations may be attempted, however with a low threshold for examination and dilatation under anaesthesia	Level of evidence III Strength of recommendation: Conditional, *for* Level of agreement: 100%

Perianal rash or skin excoriation is common after pull-through surgery, particularly among children who have had a stoma before the pull-through, long segment colonic or total colonic aganglionosis, and patients who have undergone surgery as neonates [58]. Preventive strategies involve active perineal nursing and the use of barrier ointments such as petroleum jelly, zinc-ointment, or other protective non-irritant creams. Topical antimicrobials may be also required if bacterial or fungal infection occurs.

Anastomotic strictures are a potential complication in colorectal surgery when there is a low circular anastomosis, as in ERP. Risk factors include ischemia, anastomotic leakage, and anastomotic tension. Anastomotic strictures may be less frequent after Duhamel procedure than ERP, where the reported occurrence is up to 10.6% (range: 0–18.9%) [59]. Calibration of the coloanal anastomosis at least once is advisable at around 2–3 weeks after pull-through surgery; Hegar size 12 is appropriate for infants from term up to 6 months of age. Some surgeons within ERNICA performed calibration checks infrequently during follow-up, whereas others felt that a single calibration was sufficient. There is no evidence to suggest that routine anal dilatation programs after pull-through surgery prevent strictures or enterocolitis [60, 61]. For an anastomotic stricture, a course of gentle once or twice daily anal dilatations may be attempted, however maintaining a low threshold for examination and dilatation under anesthesia.

### Recommendations for long-term follow-up (Table 6)

Structured follow-up to adulthood, including transition of care is indicated in HSCR [62–66]. As bowel dysfunction is most common during the first few years after surgery [23], patients should be monitored more closely for the early detection of problems, including defective continence, enterocolitis and late complications. Clinical follow-up should be maintained even if patients are doing well. Among patients with HSCR and a syndrome associated with cognitive impairment, reaching continence is often significantly delayed. In patients with poor functional outcomes, adequate follow-up permits the institution of interventions before school age. This is important to prevent social discrimination, and to allow participation in normal childhood activities with minimal limitations from the condition [62]. As HSCR is a rare disorder in general practice, patients should also be managed by specialized medical personnel familiar with the disease [67], and receive instructions on whom to contact and where to seek medical attention in both elective and emergency circumstances. The availability of specialist nurses improves communication and access to care, and may reduce hospitalizations/admissions and health care costs [68].

**Table 6:** Recommendations for long-term follow-up

**Children with HSCR should receive regular follow-up to adulthood within the context of an interdisciplinary care team, led by a pediatric surgeon** • Follow-up should be more frequent during 1st year of life, but regular contact should be maintained 1–2 yearly thereafter • Opportunities to engage multidisciplinary resources, including gastroenterologists, nutritional therapists, psychologists, physiotherapists, specialist nurses and social workers should be available • Attention to development of wider areas of social functioning, including self-efficacy, coping skills and sexual functioning should be addressed • Growth, nutrition and development should be followed	Level of evidence III Strength of recommendation: Strong, *for* Level of agreement: 100%
**Access to care and specialist consultation should be available** • Patients should have a named surgeon in charge of their care and clear information about where and how they should attend follow-up, including in adulthood • Instructions for where to seek emergency care should also be clearly defined • Patient support organizations networks are active in many countries for information and peer support on lived experience of the disease	Level of evidence III Strength of recommendation: Strong, *for* Level of agreement: 100%
**The introduction to adult medical disciplines should be prepared well before transition** • Discussions concerning long-term follow-up should be initiated around adolescence/puberty, and individualized care plans involving the appropriate disciplines should be formulated • Maintaining continuity and consistency of the health care into adulthood is very important to patients • Patients should be given sufficient information and increasingly engaged in decision-making regarding their healthcare with age • The future care provider should be clearly identified, with an opportunity for staged transition and liaison with paediatric services during the transition phase.	Level of evidence III Strength of recommendation: Strong, *for* Level of agreement: 100%

In patients with HSCR, life events that necessitate follow-up include puberty and sexual development, procreation and heritability, and transition to adulthood and adult care [64, 69, 70]. Psychological factors to be addressed include self-efficacy, social functioning and coping skills for residual symptoms [71, 72]. As the parents of chronically ill children also experience considerable stress, strategies to reduce psychological morbidity that also involve the parents are likely to be beneficial [62]. Achieving optimal outcomes requires collaboration between medical specialists, nurses and auxiliary resources, including psychologists and sexual therapists, physical and nutritional therapists. In addition to pediatric surgeons and gastroenterologists, urologic, gynecologic and genetic consultation should be sought as appropriate. Active involvement of the patient/family at all stages of follow-up is important to enable understanding the care plan, and to establish mutually agreed priorities for care [73]. For peer support on lived experience of the disease, patient support organizations encourage contact from patients and their families for advice and networking.

During the care pathway, as capacity for understanding increases, patients should be encouraged to acquire health literacy skills and gradually assume responsibility for their own care. Adequate information provision is important in this regard, so that including issues relating to sexual health during adulthood can be addressed [62]. Planning transition of care should commence sufficiently early, around 13–14 years of age to allow sufficient time for adjustment and should have sufficient flexibility to allow overlap and contact between the existing and future practitioners until patients are satisfactorily established in adult care [74].

### Recommendations for Hirschsprung’s-associated enterocolitis (HAEC; Table 7)

HAEC is the most frequent serious complication after pull-through surgery for Hirschsprung disease. Predisposing factors include family history of HSCR, long segment disease, Down syndrome (trisomy 21) and previous episodes of HAEC [75]. Approximately 30% of HSCR patients have at least one episode of postoperative HAEC. Although the definition remains imprecise, using the Pastor et al. HAEC score items (Table 8) [76], with a cut-off of > 4 points to identify suspected HAEC is more sensitive and may have better clinical applicability [75] than Pastor and co-workers' original cut-off of > 10 points. HAEC should be suspected in the presence of diarrhea with explosive, foul-smelling or bloody stool, and/or fever. Patients with explosive diarrhea and decreased peripheral perfusion, lethargy, and/or, dilated loops of bowel on abdominal radiographs have severe HAEC and should be admitted [75].

**Table 7:** Recommendations for Hirschsprung’s-associated enterocolitis (HAEC)

**HAEC should be clinically suspected in the presence of diarrhoea with explosive, foul-smelling stool, and/or** **>** **4 points from the Pastor et al. HAEC score items** (Table 8) [76] • The definition of HAEC remains imprecise even based on current understanding • A cut-off of > 10 points has a reported sensitivity of 42% and specificity of 100% for HAEC [75] • A cut-off of > 4 points has a reported sensitivity of 84% and specificity of 98% for HAEC [75]	Level of evidence III Strength of recommendation: Strong, *for* Level of agreement: 100%
**In suspected HAEC, there should be a low threshold for hospital admission** • In mild symptoms with no fluid or electrolyte balance disturbance and normal inflammatory markers, outpatient treatment with oral hydration +/− oral metronidazole and rectal irrigations may be appropriate, but prompt admission is indicated if symptoms do not improve. Recovery should be followed up. • Admit all other cases for in-patient monitoring and treatment • Young age (< 1 year) lowers the threshold for admission	Level of evidence III Strength of recommendation: Strong, *for* Level of agreement: 100%
**Following admission to hospital, patients with HAEC should be treated with intravenous fluid resuscitation, intravenous broad-spectrum antibiotics and rectal washouts.** • Saline rectal washouts to decompress the bowel should be performed 2–3 times per day until the patient is well enough for discharge • Antibiotics may be changed to oral metronidazole once sufficient clinical improvement occurs • Vital functions, fluid and electrolyte balance, including urine output, should be closely monitored. • Abdominal plain film x-ray should be considered • Consulting the colorectal surgical team responsible for the patient’s care is recommended • Consult intensive care unit as appropriate	Level of evidence III Strength of recommendation: Strong, *for* Level of agreement: 100%
**Intersphincteric botulinum toxin injections are recommended for patients with recurrent or persistent symptoms of outlet obstruction and/or HAEC** • In reports, 62–89% of HSCR patients with HAEC and/or outlet obstruction improved after the first botulinum toxin injection [82–86] • Injections may need to be repeated 3–6 monthly • The tendency to HAEC reduces over time; most episodes usually occur within the first few years after pull through **Principles of botulinum toxin administration** • Botox should be injected under a short general anaesthesia • The patient is positioned in lateral decubitus or lithotomy position • Injections are given in the four quadrants at the level of the dentate line into the anal sphincter musculature • Exposure of the dentate line with retractors, and/or ultrasound guidance can facilitate correct localization of the injections	Level of evidence III Strength of recommendation: Conditional, *for* Level of agreement: 100%
**Prophylactic antibiotics may be considered for patients with frequently recurring or persistent HAEC** • Antibiotics may be effective treatment of HAEC in individual patients, but it has not been shown that prophylactic antibiotics prevent recurrent HAEC. • Recurrent courses of antibiotics interfere with the long-term composition of the gut microbiota, and therefore rationalized use based on severity of symptoms is indicated	Level of evidence III Strength of recommendation: Conditional, *for* Level of agreement: 100%
**At present, there is insufficient evidence to support recommending the routine use of probiotics for the prevention of HAEC** • Although intestinal dysbiosis has been shown to be of importance in the aetiology of HAEC, there are only two randomized controlled studies of probiotics and HAEC in the literature, showing conflicting results.	Level of evidence I-III Strength of recommendation: Conditional, *against* Level of agreement: 100%
**In children with recurrent HAEC, consultation with a gastroenterologist and endoscopy should be considered** • Patients with HSCR have an increased risk of developing inflammatory bowel disease • Fecal calprotectin is a non-invasive measure of intestinal inflammation in acute and chronic enterocolitis	Level of evidence III Strength of recommendation: Conditional, *for* Level of agreement: 100%

**Table 8:** Pastor et al. (2009) HAEC score items [76]

History	
Diarrhea with explosive stool	2
Diarrhea with foul-smelling stool	2
Diarrhea with bloody stool	1
Previous history of enterocolitis	1
Physical examination	
Explosive discharge of gas and stool on rectal examination	2
Distended abdomen	2
Decreased peripheral perfusion	1
Lethargy	1
Fever	1
Radiologic examination	
Multiple air fluid levels	1
Dilated loops of bowel	1
Sawtooth appearance with irregular mucosal lining	1
Cut off sign in rectosigmoid region with absence of distal air	1
Pneumatosis	1
Laboratory	
Leukocytosis	1
Shift to left	1

Note: Using a cut-off of > 4 points to identify suspected HAEC is more sensitive and may have better clinical applicability than Pastor and co-workers' original cut-off of > 10 points [75]

Management with rectal irrigations and close observation at home with a low threshold for oral metronidazole may be an option for carefully selected, clinically well patients with mild symptoms and normal fluid balance. Differentiation from simple viral gastroenteritis may be difficult. Patients with severe HAEC should receive rectal washouts, intravenous fluid resuscitation and intravenous broad-spectrum antibiotics in hospital. Consultation of a specialist paediatric colorectal surgeon on admission, and follow-up after admission is strongly recommended. Antibiotics may be changed to oral metronidazole when sufficient clinical improvement occurs, and rectal washouts 2–3 times per day should be performed until the child is well enough for discharge. Rectal washouts should be continued at home in cases of recurrent HAEC [77]. In recurrent or persistent HAEC accompanied by symptoms of outlet obstruction (defecation difficulties, requiring assistance with e.g. a rectal tube to pass motions), mechanical obstruction or residual aganglionosis should be ruled out [78]. Contrast enemas should not be performed during an acute episode owing to the risk of perforation [79].

Intrasphincteric botulinum toxin injections [80–82] reduce the incidence of HAEC or symptoms of outlet obstruction in 62–89% of HSCR patients after the first injection [81, 83–86]. The number of hospitalizations for obstructive symptoms, particularly enterocolitis, are reduced by botulinum injection treatment [84]. Botulinum toxin injections may need to be repeated after 3–6 months, as the effects decrease over time. In rectosigmoid HSCR, the tendency to HAEC reduces over time, and most episodes occur during the first few years after pull-through surgery [23].

Although the etiology of HAEC is not fully known, intestinal dysbiosis may be an important factor [87]. The gut microbiota composition of patients with HSCR may be significantly altered compared to healthy controls. Dysbiosis in HSCR has been characterized by a lack of microbial richness, and pathologic expansions of certain taxa, particularly Enterobacteria and Bacilli [87–90], and an altered *Candida* community [87]. Although antibiotics may be effective for treating HAEC in individual patients, it has not been shown that prophylactic antibiotics reduce the incidence of recurrent HAEC. This is likely to relate to the multifactorial etiology of HAEC in HSCR, which often includes concurrent obstruction. As recurrent courses of antibiotics may also interfere with the long-term composition of the gut microbiota [90], rationalized use based on the severity of symptoms is indicated.

It has been hypothesized that the prophylactic administration of probiotics after pull-through might decrease the incidence of HAEC. However, the only two randomized controlled trials to date have shown conflicting results. In one, no risk reduction was suggested [91], but the second suggested that probiotics significantly decreased the incidence of HAEC [92]. A recent systematic review and meta-analysis included three additional studies that could not show that probiotics prevent HAEC [93]. On this basis, there is currently insufficient evidence to recommend the routine use of probiotics for the prevention of HAEC. Recent studies have showed that patients with HSCR have an approximately 5-fold increased risk of developing inflammatory bowel disease compared to the general population [94]. Endoscopic surveillance should be considered if inflammatory bowel disease is suspected, particularly if faecal calprotectin is continuously elevated.

### Management of patients with poor outcomes (Table 9)

Although surgery is effective in most cases and techniques have improved over recent years, a small proportion of patients still experience poor functional outcomes [28, 95, 96]. These can be grossly divided into faecal incontinence and obstructive symptoms, including severe constipation. In children with normal intellectual development, In children with normal intellectual development, if adequate social continence for stool has not been achieved by the age of 4 after properly conducted surgery, further evaluation is advisable [97, 98]. Management protocols should not only aim for functional improvement, but also regard the prevention of the psycho-social consequences of fecal incontinence as an important goal [72].

**Table 9:** Recommendations for management of patients with poor functional outcomes

**Children with normal intellectual development who are not continent of stool by 4 years of age should be considered for further evaluation. This should include:** • A stooling history and stooling pattern to evaluate for tendency to constipation or diarrhoea (for treatment, see below), and involuntary passage of flatus. • Dietary history and growth • Examination under anaesthesia +/− anorectal manometry to assess the integrity of the anal canal, sphincter complex and dentate line, and for the presence of rolled muscle cuff, stricture or rectal spur • Contrast enema to evaluate whether there is colonic dilatation, rectal spur, constipation or a twisted pull-through • +/− Endorectal ultrasound to assess for sphincter defects	Level of evidence III Strength of recommendation: Strong, *for* Level of agreement: 100%
**The management of fecal incontinence should aim for age-appropriate continence in children with normal intellectual development** • Primary prevention of the social consequences of fecal incontinence is a key goal of treatment • Enabling normal social integration, school attendance and ability to participate in recreational activities from the outset is important for self-esteem, friendships and long-term quality of life • Deficient fecal continence in a child is also a source of stress for caregivers and psychological support should be available for patients and families • Cognitive impairment is associated with delays in achieving voluntary bowel control	Level of evidence III Strength of recommendation: Strong, *for* Level of agreement: 100%
**Patients with an intact anal canal and appropriate pull-through but fecal incontinence should receive medical management as the first-line treatment** • For patients with a dilated colon and constipation (hypomotility), oral laxatives +/− a short course of enemas to ensure regular and complete colonic emptying • For patients without colonic dilatation and a tendency to loose stools (hypermotility), a constipating diet +/− loperamide +/− bulking agents (pectin, psyllium) • Measure fecal calprotectin, consider ileo-colonoscopy and repeat rectal biopsy • Proceed to bowel management if there is failure to respond, despite adequate dosing and compliance	Level of evidence III Strength of recommendation: Conditional, *for* Level of agreement: 100%
**Patients with fecal incontinence and damaged anal canal should receive bowel management** • Maintaining an intact anal canal is a central goal in all standard operations for HSCR, and an indication for performing pull-through surgery in specialist units • An enterostomy is an option if bowel management fails to control symptoms	Level of evidence III Strength of recommendation: Conditional, *for* Level of agreement: 100%
**Children with persistent obstructive symptoms following pull-through surgery should undergo further evaluation and treatment:** • Rectal examination and contrast enema to rule out a mechanical cause and to assess for colonic dilatation • If no mechanical cause is found, a trial of intersphincteric botulinum toxin injections • Review the histology of the proximal margins of the originally resected bowel • Repeat rectal biopsies to ensure normal innervation of the pulled-through bowel • If repeated botulinum toxin injections are ineffective, histology is normal and there is no mechanical cause, bowel management can be offered • Consider re-do surgery in patients with a recalcitrant stricture, twisted pull-through, rolled muscle cuff (Soave), rectal spur (Duhamel) or transition zone pull-through	Level of evidence III Strength of recommendation: Strong, *for* Level of agreement: 100%
**Bowel management programme should comprise individualized care based on the symptom profile, local recommendations and values/preferences of the patient/carer(s)** • The goal of bowel management is to achieve regular and complete colonic emptying at predictable intervals • Options include regular retrograde enemas or antegrade colonic irrigation via an antegrade continence enema appendicostomy (ACE) or cecostomy +/− dietary modifications +/− laxatives • Psychological support can assist patients and families in coping with symptoms • An enterostomy may be required in isolated cases for intractable symptoms	Level of evidence III Strength of recommendation: Conditional, *for* Level of agreement: 100%

The initial evaluation comprises a full survey of the stooling pattern, dietary history, growth and development. Examination of the integrity of the dentate line and sphincter muscles should be performed, preferably under general anaesthesia. This may be complemented by anorectal manometry or endorectal ultrasound [44, 99]. In faecal incontinence, contrast enemas are helpful in differentiating between hypo- and hypermotile colon. Both hypomotility and outlet obstruction may lead to overflow incontinence and colonic dilatation, and should be differentiated from hypermotility disorders because their treatment is different. In patients with an intact dentate line and good sphincter function without outlet obstruction but colonic *hypomotility*, first line management comprises oral laxatives, supplemented with a short course of enemas if required. These aim to ensure complete colonic emptying to prevent faecal incontinence and soiling due to overflow. Patients with an intact dentate line and good sphincters but colonic *hypermotility*, most commonly due to loss of rectal reservoir, need interventions to slow down colonic transit. These include a constipating diet, loperamide and bulking agents (e.g. pectin, psyllium). If the dentate line and/or sphincter musculature are significantly damaged, bowel management to empty the colon by artificial means will be necessary to achieve social continence (see below) [100]. If both medical management and bowel management fail, a permanent stoma may be the last resort.

Several mechanical factors can lead to postoperative obstructive symptoms. These include an anastomotic stricture, Duhamel spur, obstructed Soave muscular cuff, twisted pull-through or retained aganglionotic or transition zone pull-through. Obstructive symptoms can occur with or without HAEC. Following a thorough clinical, dietary and stooling history as for fecal incontinence, rectal examination and contrast enema should be performed [101–105]. Histology should be reviewed, particularly with regard to the adequacy of the proximal resection margins of the pull-through [55, 101]. Repeating transanal colonic biopsies should be considered to confirm normal innervation of the pulled through colon. For anastomotic strictures, a gentle course of anal dilatations may be attempted, with a low threshold for general anaesthesia if poorly tolerated. If no mechanical cause is found, first-line treatment is intersphincteric botulinum toxin injections to relieve internal sphincter achalasia. If symptoms fail to improve after repeated (> 3) botulinum toxin injections, bowel management is the second-line choice of treatment.

Bowel management comprises individualized management based on the patient’s preferences. Options include regular retrograde enemas, or antegrade colonic irrigation via an antegrade continence enema appendicostomy (ACE) or cecostomy (CHAIT, button) in conjunction with oral laxatives and dietary modifications. Re-do surgery should be considered in symptomatic patients with a recalcitrant stricture, twisted pull-through, rolled muscle cuff (Soave), rectal spur (Duhamel), or aganglionic/transition zone pull-through. The full evaluation and re-do surgical procedure should be performed in a centre with experience of complex pathology in HSCR and re-do procedures. Although redo-surgery is appropriate in selected cases resolves obstructing symptoms, it may be associated with relatively high rates of fecal incontinence and thus, careful patient selection is important [103, 105].

### Recommendations for genetic screening (Table 10)

Most cases of HSCR that occur without an associated genetic syndrome or chromosomal anomaly are sporadic. The main gene in non-syndromic HSCR is the proto-oncogene RET. Coding mutations, which usually cause a loss of function in RET are present in approximately 15–35% of sporadic cases [106–114]. In patients with a family history of HSCR, the occurrence of RET coding mutations is much higher, up to 50% [108, 109, 111]. The second most commonly mutated gene in non-syndromic HSCR is EDNRB (endothelin receptor-beta) which is affected in ~ 5% of cases [112]. Besides RET and EDNRB, many other genes have been implicated in HSCR, but the chances of detecting a mutation in these genes are very low. Although most HSCR is found in isolation it also is seen in the context of several syndromes, including Down syndrome (Trisomy 21), which is the most common, Shah-Waardenburg syndrome, Congenital Central Hypoventilation syndrome (CCHS), Mowat-Wilson syndrome, and Goldberg-Shprintzen syndrome.

**Table 10:** Recommendations for genetic screening of patients with HSCR

**In non-syndromic HSCR, genetic testing of*RET*should be considered** • RET remains the major gene in HSCR • Molecular testing allows a more accurate estimation of the risk of recurrence • Genetic testing of RET allows exclusion of the rare possibility of MEN 2A-associated RET mutation that is associated with an increased risk of medullary thyroid cancer • Parents or patients who wish to have genetic screening should be offered referral for genetic consultation • Genetic consultation is also recommended for patients with a family history of HSCR, where the incidence of RET mutations is even higher	Level of evidence II-III Strength of recommendation: Conditional, *for* Level of agreement: 100%
**In syndromic HSCR, patients should be offered referral for genetic consultation and screening for the specific gene associated with the syndromic phenotype**	Level of evidence III Strength of recommendation: Conditional, *for* Level of agreement: 100%

Although most mutations in RET are inactivating, activating RET mutations are present in approximately 2–3% of all sporadic HSCR cases [113]. These mutations can cause multiple endocrine neoplasia type 2A (MEN2A), a cancer syndrome characterized by medullary thyroid carcinoma, phaeochromocytoma of the adrenal glands, and hyperplasia of the parathyroid glands [114, 115]. In non-syndromic HSCR, referral for genetic testing of *RET* to exclude the rare possibility of a MEN 2A associated *RET* mutation that is also associated with an elevated risk for MTC should therefore be considered [113]. Molecular testing outcomes may also give a more accurate estimation for the parents of a HSCR patient of the risk of recurrence in future pregnancies. As many RET mutations prove to be sporadic and have not been inherited from the parents, screening is preferably performed in a TRIO setting that involves testing the father, mother and child. In suspected syndromic HSCR, patients should be screened for the gene that is associated with that specific phenotype.

#### Evidence gaps and targets for further research

There remain many caveats in the knowledge of Hirschsprung’s disease. There are currently no practical capabilities for the prenatal diagnosis of HSCR, as abnormal sonographic findings are absent in the majority of fetuses with HSCR [116]. The definitions, causes and predisposing factors for HAEC and bowel dysmotility in the remaining ganglionic bowel remain incompletely understood. The composition intestinal microbiota and gut mucosal immunity in the pathogenesis of HAEC is a topic of current research interest. With regard to operative management, more comparative studies of the predominant pull-through techniques (Duhamel and ERP) are needed to gather higher quality evidence of the long-term outcomes, and whether laparoscopy-assisted techniques confer advantages. Little is also known about the sexual function and fertility after pull-through surgery, although physical sexual functions appear to be preserved in the majority [117]. However, it has been shown that technically comparable low pelvic surgery for other pediatric bowel problems, impacts on later fertility, especially in females [118]. Further prospective research and multicenter studies are needed to obtain a full understanding of the long-term implications of HSCR on patients’ health.

## Conclusions

In rare or low-prevalence diseases such as HSCR, there is limited availability of high-quality clinical evidence. However, patients born with these conditions continue to require highly specialized care from infancy up to adulthood. In this document, consensus-based guidelines that describe the preferred approach of ERNICA, an international panel of experts from 10 major European centres to the management of rectosigmoid HSCR are presented. ERNICA remains committed to upholding and improving care standards for patients with HSCR.

## Materials and methods

### Membership of the guideline workgroup

The workgroup comprised an international panel of experts in HSCR from specialist centres in 8 European countries (Denmark, France, Germany, Finland, Italy, Netherlands, Norway, Sweden and the United Kingdom). These included pediatric surgeons, histopathologists, gastroenterologists, microbiologists and geneticists from 10 ERNICA (ERN) member centres and 3 associated experts selected by ERNICA. Patient representatives from SoMA, the German patient support organisation for anorectal malformations and HSCR and A.Mor.Hi, the Italian Society for HSCR, participated throughout the process.

Accreditation as an ERN centre requires demonstration and approval of specific specialist competencies and compliance with a comprehensive set of operational criteria [119]. These include demonstration of quality in organizational infrastructure, patient-centered management, clinical governance, sufficient care volumes, ability to report outcomes and commitment to upholding expertise through research, training and education. Recognition of competencies and membership is based on regular internal and external assessment.

### Background preparation

A literature search was conducted using PubMed of articles published on HSCR from 1990 to 2018, and their references to identify articles that had not been brought up by the original search. Where studies of HSCR or pediatric populations were scarce or unavailable, data was derived from relevant studies from adult populations. Within groups, expert clinicians selected those publications they considered most relevant and highest quality for the reference pool of the guideline. Patient representatives contributed publications that best reflected their values to the workgroup. All authors had access to the reference pool during development. Articles were assigned an evidence level of I-III based on the Canadian Task Force on the Periodic Health Examination classification system (1979) summarized in Table 11 [120], with the expectation that in rare or low prevalence diseases most would fall into category III (descriptive studies, expert opinion).

**Table 11:** Grading of levels of Evidence [120]

**Level of Evidence**	
• I Evidence from at least one randomized controlled trial	
• II1 Evidence from well-designed case-control or cohort studies, preferably from more than one research group or centre	
• II2 Comparisons between times and places with or without the intervention, or dramatic results of uncontrolled experiments	
• III Opinions of respected authorities, based on clinical experience, descriptive studies or reports of expert committees.	

### Determination of the core recommendation statements

The working group met on three occasions: 1) to discuss the content of the guideline, 2) to decide on the core recommendations and supporting statements, and 3) to finalize the content, including voting on levels of agreement. After drafting recommendations, the supporting text and summary of the evidence was prepared. The AGREE II framework was used to assess the scope and rigour of the methodology [121]. The GRADE approach was used to rate the strength of the recommendations [122, 123]. Updating of the recommendations at 5-year intervals by ERNICA was planned.

### Assigning strength of the recommendations

Recommendations were classed as *strong* (for or against), if the workgroup felt highly confident of the balance between desirable and undesirable consequences [122, 123]. Recommendations were classed as *conditional* (for or against), if the appropriate course of action was considered subject to patient values and preferences, availability of resources and/or setting of the intervention [122, 123].

### Voting on levels of agreement

Voting on levels of agreement was conducted at the final meeting. Recommendations receiving < 100% agreement were further discussed, revised and voted on again at the meeting until consensus was reached; > 75% agreement was considered necessary to finalize recommendations [124].

## Data Availability

Not applicable to article type.

## Abbreviations

ACE: Antegrade continence enema
AChE: Acetylcholine esterase
EDNRB: Endotheln receptor beta
ERN: European Reference Network
ERNICA: The European Reference Network for for rare inherited and congenital digestive disorders
ERAS: Enhanced recovery after surgery
ERP: Endorectal pull-through
EU: European Union
HSCR: Hirschsprung’s disease
H&E: Haematoxylin and eosin
MEN: Multiple endocrine neoplasia
MTC: Medullary thyroid cancer
RET: Rearranged during transfection proto-oncogene
